# Ammonia-oxidizing bacteria dominate active ammonia oxidation during nitrogen fertilizer-induced acidification of a maize-cultivated black soil

**DOI:** 10.1093/ismeco/ycag285

**Published:** 2026-10-14

**Authors:** Yuxiang Song, Xiangxin Sun, Yuanfeng Cai, Weiwei Cao, Yufang Wang, Zhongjun Jia

**Affiliations:** State Key Laboratory of Soil and Sustainable Agriculture, Institute of Soil Science, Chinese Academy of Sciences, Nanjing 211135, Jiangsu, China; University of Chinese Academy of Sciences, Beijing 100049, China; Soil Ecology Lab, College of Resources and Environmental Sciences, Nanjing Agricultural University, Nanjing 210095, Jiangsu, China; State Key Laboratory of Soil and Sustainable Agriculture, Institute of Soil Science, Chinese Academy of Sciences, Nanjing 211135, Jiangsu, China; University of Chinese Academy of Sciences, Beijing 100049, China; State Key Laboratory of Soil and Sustainable Agriculture, Institute of Soil Science, Chinese Academy of Sciences, Nanjing 211135, Jiangsu, China; University of Chinese Academy of Sciences, Beijing 100049, China; Institute of Biology, Gansu Academy of Sciences, Lanzhou 730000, Gansu, China; State Key Laboratory of Soil and Sustainable Agriculture, Institute of Soil Science, Chinese Academy of Sciences, Nanjing 211135, Jiangsu, China; University of Chinese Academy of Sciences, Beijing 100049, China; State Key Laboratory of Black Soils Conservation and Utilization, Northeast Institute of Geography and Agroecology, Chinese Academy of Sciences, Changchun 130102, Jilin, China

**Keywords:** nitrification, soil acidification, ammonia oxidizers, *Nitrosospira*, acid tolerance, microbial aggregation

## Abstract

Nitrogen fertilization accelerates soil acidification by stimulating ammonia oxidation, yet the dominant active ammonia oxidizers and their acid-adaptation strategies remain unresolved. We combined DNA stable isotope probing (DNA-SIP) with enrichment cultivation, metagenomics, and metatranscriptomics to identify active ammonia oxidizers and characterize acid-tolerance responses in a maize-cultivated black soil from Northeast China. Repeated urea and NH_4_Cl additions stimulated nitrification and acidified the soil, with pH decreasing from 7.35 to 4.73 under NH_4_Cl addition. DNA-SIP showed that ammonia-oxidizing bacteria (AOB), rather than ammonia-oxidizing archaea (AOA), dominated active ammonia oxidation during acidification. Phylogenetic analysis of *amoA* sequences from isotope-labelled higher-density DNA fractions identified *Nitrosospira*-related D11 and D12 clusters among active AOB. An acid-tolerant enrichment culture retained nitrification activity at pH 5.0 and yielded a D11-affiliated *Nitrosospira* MAG, designated *Nitrosospira* sp. NT. Its *amoA* sequence showed 98.2% nucleotide identity to a SIP-derived D11 *amoA* sequence, linking the enrichment-derived population to an active soil D11 cluster. Under acidic conditions, the enrichment culture showed pronounced aggregation with increased fungal occurrence, suggesting that fungal occurrence may be associated with the aggregated phenotype. Genomic and transcriptomic analyses indicated coordinated responses involving substrate acquisition and acid buffering, respiratory energy metabolism, intracellular pH homeostasis, envelope stress responses, and candidate systems potentially related to surface-associated growth and stress responses. These findings link community-level SIP evidence with genomic and transcriptional insights from an enrichment-derived representative of an active D11 cluster and show that acid-tolerant AOB can dominate active ammonia oxidation and sustain nitrification during nitrogen fertilizer-induced acidification in the black soil examined.

## Introduction

Soil acidification, largely driven by the extensive application of synthetic nitrogen fertilizers, has profound impacts on nitrogen cycling processes in terrestrial ecosystems [1, 2]. The application of ammonium-based fertilizers stimulates ammonia oxidation and generates substantial proton release, thereby accelerating soil acidification [1, 3]. However, acidification can subsequently suppress ammonia oxidation itself [4, 5], creating a dynamic feedback between nitrification and soil acidity. Ammonia oxidation is mediated by ammonia-oxidizing archaea (AOA), ammonia-oxidizing bacteria (AOB), and complete ammonia-oxidizing (comammox) *Nitrospira* [6]. Ammonia availability and pH are recognized as major determinants of niche differentiation among ammonia oxidizers, particularly between AOA and AOB [7–10]. Yet, during agricultural soil acidification, pH progressively decreases under sustained nitrogen inputs and fluctuating ammonia availability [2], conditions that may continuously reshape ammonia oxidizer activity and competitive interactions. Consequently, although the ecological distributions of ammonia oxidizers in acidic soils have been increasingly investigated, whether AOA or AOB remain active and sustain ammonia oxidation during progressive fertilizer-induced soil acidification remains poorly resolved.

AOA are generally considered better adapted to acidic and oligotrophic environments owing to their high ammonia affinity and putative acid tolerance [11–14], and they frequently dominate autotrophic ammonia oxidation in acidic and low-nitrogen soils [8, 15, 16]. In contrast, AOB are typically associated with neutral to alkaline soils and high ammonia availability [17–19], where they often support elevated nitrification activity in fertilized agricultural systems [20]. This ecological partitioning has long shaped the classical view that AOA dominate acidic soils, whereas AOB prevail under nitrogen-rich conditions. However, accumulating evidence challenges this framework. DNA stable isotope probing (DNA-SIP) studies have reported substantial AOB activity in acidic forest and paddy soils [21, 22], while the cultivation of acid-tolerant AOB strains demonstrates that AOB are not necessarily restricted to circumneutral environments [23, 24]. These findings suggest that the functional partitioning of AOA and AOB may be more nuanced than traditionally thought, particularly in agricultural soils undergoing nitrogen fertilizer-induced acidification.

DNA-SIP has become a powerful approach for identifying active ammonia oxidizers under different environmental conditions [25, 26], while strain-based enrichment cultures provide valuable insights into the physiological and metabolic traits of specific ammonia oxidizers [11, 27]. Compared with AOA, for which potential acid-tolerance mechanisms have been explored [14], the mechanisms underlying acid tolerance in AOB remain poorly understood. To date, two representative acid-tolerant γ-AOB, *Ca*. Nitrosoglobus terrae TAO100 and *Ca*. Nitrosacidococcus tergens RJ19 have been enriched and demonstrated to grow at pH 5.0 and 2.5, respectively [23, 24]. Biofilm formation, urea utilization, and cation transporters have been proposed as potential contributors to their growth under acidic conditions [23, 24]. In β-AOB, DNA-SIP studies have revealed their important contribution to nitrification in acidic soils [21, 22], and the recent enrichment of three *Nitrosospira* strains, TNL100, TNL200, and TNL300, capable of growth at pH 6.0, has further advanced our understanding of acid-tolerant physiology in β-AOB [28]. Nevertheless, the metabolic strategies by which AOB adapt to acidic environments remain insufficiently understood.

Northeast China, one of the world’s major black soil regions and a key maize-producing area in China, plays a critical role in national agricultural production [29, 30]. However, long-term intensive fertilization, particularly nitrogen fertilization, has accelerated soil acidification, resulting in a substantial decline in soil pH [31]. Identifying the ammonia oxidizers that remain active as agricultural soils acidify is important because ammonia oxidation initiates nitrification and contributes directly to proton production; its persistence may therefore sustain nitrogen transformation while further promoting soil acidification. Moreover, following ammonia oxidizer activity during progressive acidification can reveal ecological responses that may not be evident from studies of chronically acidic soils alone. We hypothesized that sustained nitrogen fertilization would selectively favor acid-tolerant AOB capable of maintaining ammonia oxidation as soil pH declines and that these AOB would exhibit physiological and transcriptional responses associated with adaptation to acidic conditions.

In this study, we established DNA-SIP microcosms using maize-cultivated black soil to identify ammonia oxidizers that remained active under sustained nitrogen input and progressive acidification. We further enriched acid-tolerant ammonia oxidizers and integrated metagenomic and metatranscriptomic analyses to characterize their metabolic potential and transcriptional responses under acidic conditions. This integrated approach allowed us to determine which ammonia oxidizers drive nitrification during nitrogen fertilizer-induced acidification and to explore physiological and transcriptional responses associated with acid tolerance in acidifying agricultural soils.

## Materials and methods

### Soil sampling and characterization

Soil samples were collected from a maize field at the Lishu Conservation Tillage Research and Development Station of the Institute of Applied Ecology, Chinese Academy of Sciences, located in Jilin Province, Northeast China (43°19′ N, 124°14′ E). The soil at the site is classified as black soil, a typical Mollisol widely distributed in Northeast China. The field has been under continuous maize cultivation for many years. Samples were collected from the top 0–20 cm soil layer. One portion of each sample was air-dried and sieved for physicochemical analysis, while another portion was stored at −20°C until incubation and sequencing. The soil had a pH of 7.35, with 22.33 g kg^−1^ soil organic matter, 12.95 g kg^−1^ soil organic carbon, 1.44 g kg^−1^ total nitrogen, 130.01 μg g^−1^ hydrolyzable nitrogen, 0.77 g kg^−1^ total phosphorus, 35.19 μg g^−1^ available phosphorus, 23.10 g kg^−1^ total potassium, and 167.96 μg g^−1^ available potassium. The near-neutral soil was selected to allow the progression of nitrogen fertilizer-induced acidification and the accompanying changes in ammonia-oxidizing activity to be examined from a non-acidified starting condition.

### Soil microcosms for stable isotope probing of active ammonia oxidizers

Fresh soil equivalent to 5.0 g dry weight was added to 120 ml serum bottles, adjusted to 60% of the maximum water-holding capacity, sealed with butyl rubber stoppers, and pre-incubated at 28°C in the dark for 10 days. The headspace of each bottle was amended with 5% (v/v) isotopically enriched ^13^C-CO_2_ or ^12^C-CO_2_ (Shanghai Research Institute of Chemical Industry, Shanghai, China), in the presence or absence of 100 Pa acetylene (C_2_H_2_), an inhibitor of ammonia monooxygenase-containing microorganisms. To maintain oxic conditions, bottles were flushed weekly with pressurized synthetic air (20% O_2_, 80% N_2_) for 1 min and resupplied with ^13^C-CO_2_ or ^12^C-CO_2_. The soil microcosms received weekly applications of 100 μg urea-N or NH_4_Cl-N per gram of dry soil. For isotope-labelled SIP microcosms, the urea treatment received >99 atom% ^13^CO_2_ and >99 atom% ^13^C-urea, whereas the NH_4_Cl treatment received >99 atom% ^13^CO_2_ and >99 atom% ^15^N-NH_4_Cl. These isotope combinations were selected to enhance isotopic enrichment of DNA from metabolically active ammonia oxidizers. The supplied ^13^CO_2_ served as a tracer for autotrophic carbon fixation; hydrolysis of ^13^C-urea could provide an additional source of labelled inorganic carbon, whereas ^15^N-NH_4_Cl provided an additional isotope tracer through potential assimilation of NH_4_^+^-derived nitrogen. The individual contributions of ^13^C and ^15^N incorporation to DNA density shifts were not quantified separately.

Independent microcosms were established for each treatment and sampling time, with three biological replicates per treatment–time combination. The Day 0 samples were collected at the end of the 10-day pre-incubation period, before the isotope and nitrogen treatments were initiated, and served as the baseline. Microcosms assigned to Days 42 and 63 were destructively harvested at the corresponding sampling times; thus, the same bottles were not repeatedly sampled over time. Harvested soil samples were used for measurements of soil pH and inorganic nitrogen concentrations and for DNA extraction. The extracted DNA was subsequently used for *amoA* gene quantification and DNA-SIP density-gradient fractionation.

Isopycnic density-gradient centrifugation was conducted independently for DNA extracted from each biological replicate (*n* = 3), using 3.0 μg of DNA per gradient according to a previously described protocol [32]. Following density-gradient fractionation, AOB and AOA *amoA* gene abundances were quantified across the CsCl gradients for all three biological replicates (*n* = 3). For subsequent *amoA* sequencing, one biological replicate (*n* = 1) was selected for each corresponding treatment and sampling time. Higher-density DNA fractions were selected based on isotope-induced shifts relative to the corresponding ^12^C-control profiles, with CsCl buoyant-density ranges of 1.741–1.756 g ml^−1^ for AOB and 1.735–1.754 g ml^−1^ for AOA.

### Quantification of *amoA* genes

The abundances of archaeal and bacterial *amoA* genes were quantified by quantitative PCR using the primer set Arch-amoAF/Arch-amoAR [33] and amoA1F/amoA2R [34], respectively. The amplification efficiencies for all assays ranged from 88.4% to 100%, with *R*^2^ values ranging from 0.982 to 0.994. Detailed descriptions are provided in Supplementary Table S1.

### Enrichment cultivation

The enrichment culture was established from the same initial maize-cultivated black soil used for the soil microcosm experiments. Briefly, soil was used to inoculate 150 ml of AOB medium containing 25 mM (NH_4_)_2_SO_4_, 0.73 mM MgSO_4_·7H_2_O, 0.18 mM CaCl_2_·2H_2_O, 0.1 mM EDTA-NaFe, 0.54 μM CuSO_4_·5H_2_O, 43.2 mM KH_2_PO_4_, 3.89 mM NaH_2_PO_4_, 3.77 mM Na_2_CO_3_, 0.5 μM H_3_BO_3_, 0.5 μM MnCl_2_·4H_2_O, 0.8 μM CoCl_2_·6H_2_O, 0.1 μM NiCl_2_·6H_2_O, 0.5 μM ZnSO_4_·7H_2_O, and 0.15 μM Na_2_MoO_4_·2H_2_O. The medium was adjusted to pH 6.0 and buffered with 50 mM MES to provide a moderately acidic selective pressure while maintaining sufficient ammonia-oxidizing activity for long-term serial enrichment of acid-tolerant AOB. Cultures were incubated aerobically in the dark in 250-ml Erlenmeyer flasks at 28°C with shaking at 180 rpm. During incubation, medium pH and the concentrations of nitrite (NO_2_^−^), nitrate (NO_3_^−^), and ammonium (NH_4_^+^) were monitored daily. Medium pH was adjusted as required using 1 M NaOH or 1 M HCl. After most of the NH_4_^+^ had been consumed, 30% (v/v) of the culture was transferred into fresh medium for subsequent enrichment. The enrichment process was maintained for 2 years. Detailed analytical procedures for these parameters are provided in the Supplementary Materials.

To compare the enrichment phenotype under different pH conditions, the long-term pH 6.0 enrichment culture was used as the inoculum and serially propagated in the same AOB medium adjusted to either pH 6.0 or pH 8.0. At each transfer, 30% (v/v) of the culture was transferred to fresh medium after NH_4_^+^ depletion. Cultures were incubated at 28°C in the dark with shaking at 180 rpm under otherwise identical conditions. Culture appearance was visually monitored and recorded during serial propagation.

### pH-gradient nitrification assay

Nitrification activity of the enrichment culture was assessed under different pH conditions as described in the Supplementary Materials. Medium pH was monitored daily throughout the assay and readjusted to the designated pH value using 1 M NaOH or 1 M HCl as required.

### Fungal isolation and identification

To compare fungal occurrence under different pH conditions, the long-term pH 6.0 enrichment culture was serially propagated in media adjusted to pH 6.0 or pH 8.0. Fungi were isolated from the pH 6.0 and pH 8.0 enrichment cultures on potato dextrose agar (PDA) supplemented with ampicillin (150 μg ml^−1^), with uninoculated PDA plates included as negative controls. Fungal colonies recovered from the pH 6.0 enrichment culture were repeatedly purified and identified by ITS1/ITS4 amplification followed by Sanger sequencing and BLASTN analysis. Detailed procedures are provided in the Supplementary Methods.

### Fluorescence *in situ* hybridization and scanning electron microscopy

Fluorescence *in situ* hybridization (FISH) and scanning electron microscopy (SEM) analyses were performed to characterize the spatial distribution and morphology of cells in the enrichment culture. Detailed procedures are described in the Supplementary Methods.

### Metagenomic sequencing

For metagenomic sequencing, cultures were grown for 3 days to the exponential phase in 250 ml Erlenmeyer flasks containing 150 ml of medium adjusted to pH 6.0. Cells from 20 ml of culture were collected by centrifugation at 8000 rpm for 5 min, and the resulting pellets were used for DNA extraction. Three biological replicates were analyzed independently. Each sample yielded more than 32.47 Gb of clean data, giving a total of 100.74 Gb across all samples, with Q30 values above 95.29%. Details of DNA extraction and sequencing data analysis are provided in the Supplementary Information.

### Metatranscriptomic sequencing

For metatranscriptomic analysis, cultures were grown for 3 days until they reached the exponential phase in 1000 ml Erlenmeyer flasks containing 500 ml of medium adjusted to pH 6.0. Each culture was then divided into two aliquots, which were adjusted to pH 5.0 or 8.0 using 1 M HCl or 1 M NaOH, respectively, and incubated for an additional 24 h. A 24-h exposure period was selected to capture an established short-term transcriptional response to acidic conditions while minimizing potential secondary effects associated with prolonged cultivation, as previous studies have shown that ammonia oxidizers can exhibit transcriptional responses to environmental perturbations within hours to 24 h [35, 36]. Three biological replicates were prepared for each pH treatment. During this period, pH was monitored and adjusted as needed. Cells from 20 ml of culture were collected by centrifugation at 8000 rpm for 5 min, and the resulting pellets were used for RNA extraction. In total, metatranscriptomic sequencing generated 76.43 Gb of data, with more than 94.9% of bases achieving Q30. Differential expression analysis was performed using the DESeq2 package in R [37]. Genes with an absolute log_2_ fold change (|log_2_FC|) > 1 and an adjusted *P* value <.05 were considered significantly differentially expressed. Details of RNA extraction and sequencing data analysis are provided in the Supplementary Information.

### Statistical analysis

Statistical analyses were performed using SPSS version 27.0. For the soil microcosm experiments, treatment and incubation time (42 and 63 days) were analyzed as fixed factors using two-way analysis of variance (ANOVA), including the treatment × time interaction. Samples collected at the two incubation times were obtained from independent microcosms by destructive sampling and were therefore not treated as repeated measures. Pairwise comparisons among treatment × time combinations were performed using Tukey’s HSD test. The Day-0 soil represented the common pretreatment baseline and was not included in the two-way ANOVA; comparisons of individual treatment × time combinations with the Day-0 baseline were performed separately using Dunnett’s test. Differences were considered statistically significant at *P* < .05.

## Results

### Changes in physicochemical properties and ammonia oxidizers

During the microcosm incubation period, soil pH declined markedly in both the urea- and NH_4_Cl-amended microcosms, indicating sustained soil acidification. Specifically, NH_4_Cl addition induced stronger acidification, as indicated by a gradual decrease in soil pH from 7.35 to 4.73, whereas acetylene addition suppressed this pH decline (Fig. 1a). Soil nitrification was assessed based on the production of NO_x_^–^-N, defined as the sum of NO_2_^–^-N and NO_3_^–^-N (Fig. 1b). After 42 and 63 days of incubation, soil NO_x_^–^-N concentrations increased significantly in the urea- and NH_4_Cl-treated soils. In contrast, nitrification was strongly inhibited in the acetylene-amended treatments, resulting in the accumulation of NH_4_^+^-N (Fig. 1b, S1). Because acetylene is a classical inhibitor of ammonia oxidation, these results indicate that NO_x_^–^-N accumulation and the accompanying pH decline were primarily driven by nitrification itself.

**Figure 1:**
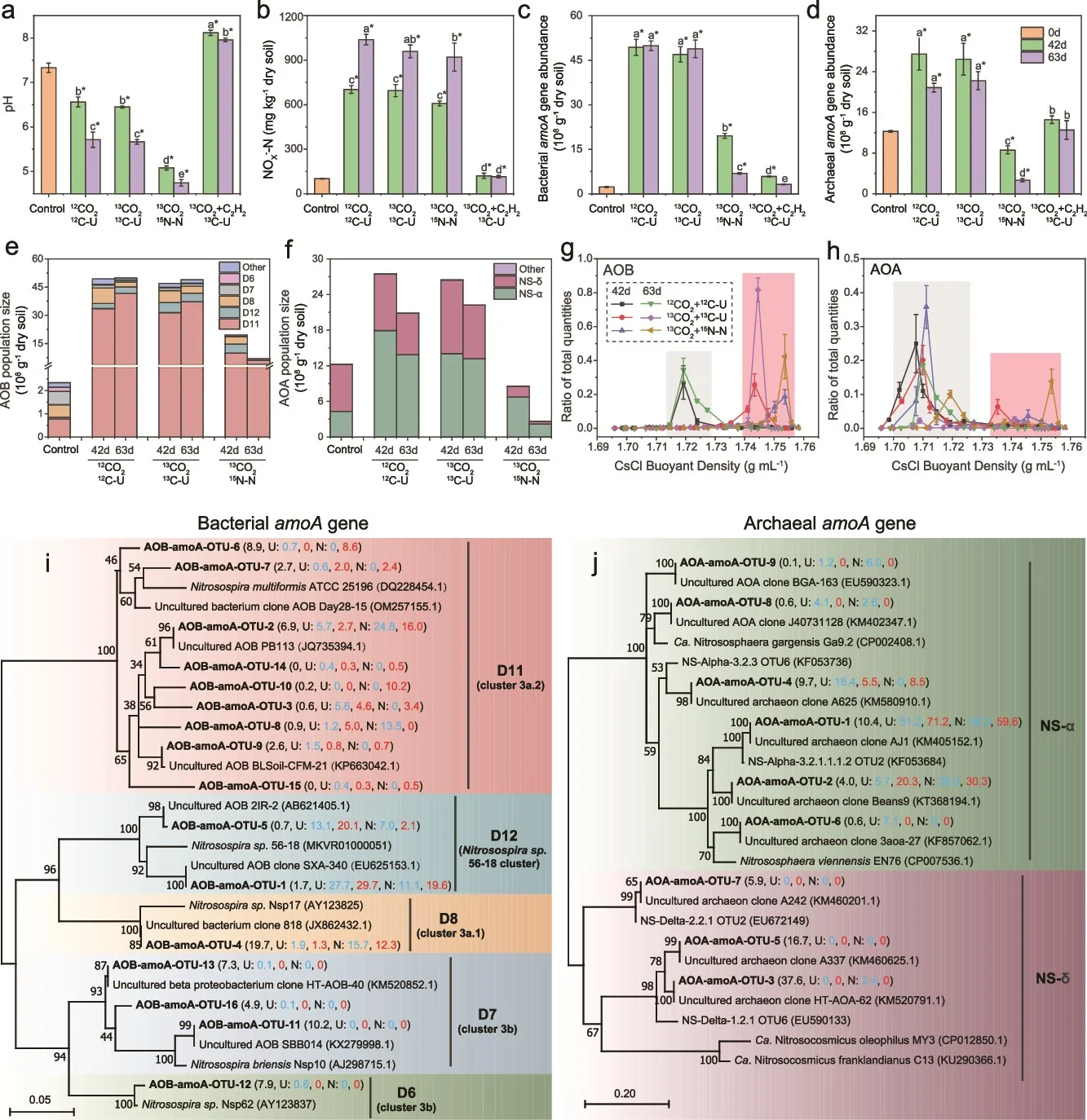
Stable isotope probing (SIP) identifies active ammonia oxidizers during soil acidification. (a–d) Changes in soil pH, NO_x_^–^-N concentration, and AOB and AOA *amoA* gene abundances during 42- and 63-day SIP incubations. ^13^C-U, ^13^C-urea; ^15^N-N, ^15^N-NH_4_Cl. Day 0 represents the soil collected at the end of pre-incubation before isotope and nitrogen treatments. (e, f) Estimated abundances of individual AOB clusters and AOA lineages, calculated from total bacterial or archaeal *amoA* gene abundance and the corresponding cluster/lineage relative abundance (Fig. S2). (g, h) Distribution of bacterial and archaeal *amoA* genes across DNA buoyant-density gradients at days 42 and 63. The ^12^C-control profiles are shown as reference distributions for evaluating isotope-induced shifts toward higher-density DNA fractions. The right-hand boxed regions indicate the higher-density DNA fractions selected for *amoA* sequencing. (i, j) Phylogenetic identification of active AOB and AOA based on *amoA* sequences from Day 0 control DNA and isotope-labeled DNA. Numbers indicate relative abundances (%) of each OTU in total soil DNA at Day 0 and isotope-labeled DNA from urea- (U) or NH_4_Cl-amended (N) soils at Days 42 and 63. For example, AOB-amoA-OTU-1 (1.7; U: 27.7, 29.7; N: 11.1, 19.6) indicates that OTU-1 accounted for 1.7% of total AOB *amoA* sequences in total soil DNA at Day 0, 27.7% and 29.7% in isotope-labeled DNA from urea-amended soil at Days 42 and 63, respectively, and 11.1% and 19.6% in isotope-labeled DNA from NH_4_Cl-amended soil at Days 42 and 63, respectively. Phylogenetic trees were constructed with IQ-TREE v1.6.12 using the best-fit models selected by Bayesian information criterion (BIC) (TVMe+I + G4 for AOA and HKY + F + I + G4 for AOB); branch support was estimated from 1000 bootstrap replicates. For panels a–d, treatment and incubation time were analyzed as fixed factors using two-way ANOVA, including the treatment × time interaction. Pairwise comparisons among treatment × time combinations were performed using Tukey’s HSD test, and different lowercase letters indicate significant differences (*P* < .05). The Day-0 soil was treated as the common pretreatment baseline and was not included in the two-way ANOVA; asterisks indicate significant differences from the Day-0 baseline based on Dunnett’s test (*P* < .05). AOB and AOA *amoA* gene abundances were log10-transformed prior to statistical analysis. For panels g and h, DNA-SIP density-gradient fractionation and *amoA* quantification were performed independently using three biological replicates (*n* = 3) for each condition. For panels i and j, *amoA* sequencing of isotope-labelled higher-density DNA fractions was performed using one biological replicate (*n* = 1) for each corresponding treatment and sampling time.

During the incubation period, AOB responded more strongly than AOA to nitrogen addition. Both urea and NH_4_Cl increased AOB *amoA* gene abundance, whereas their effects on AOA differed: urea increased AOA *amoA* gene abundance, whereas NH_4_Cl decreased it (Fig. 1c and d). In addition, AOA *amoA* gene abundance declined significantly from Day 42 to Day 63. Both urea and NH_4_Cl increased the abundances of AOB clusters D8 (*Nitrosospira* cluster 3a.1), D11 (*Nitrosospira* cluster 3a.2), and D12 (*Nitrosospira* sp. 56–18 cluster), whereas the abundances of clusters D6 (*Nitrosospira* cluster 3b) and D7 (*Nitrosospira* cluster 3b) declined (Fig. 1e). The AOA community was dominated by clades *Nitrososphaerales*-Alpha and -Delta (NS-α and -δ), with the increase in clade NS-α after incubation accounting for most of the observed increase in AOA abundance (Fig. 1f).

### Isotope labeling of active ammonia oxidizers

After ultracentrifugation of total DNA extracted from isotope-labelled and control microcosms, the distribution of *amoA* genes was quantified across DNA buoyant-density gradients, revealing isotope incorporation by actively growing ammonia oxidizers in both urea- and NH_4_Cl-amended soils (Fig. 1g and h). The ^12^C controls showed AOB and AOA *amoA* genes predominantly in lower-density DNA fractions, providing the reference profiles for assessing isotope-induced density shifts (Fig. 1g and h). After 42 and 63 days of incubation, AOB *amoA* genes were detected predominantly in DNA fractions with higher CsCl buoyant densities in both the ^13^CO_2_ + ^13^C-urea and ^13^CO_2_ + ^15^N-NH_4_Cl treatments. The proportion of AOB *amoA* genes in these higher-density fractions further increased from Day 42 to Day 63 (Fig. 1g). AOA *amoA* genes also showed a shift towards higher-density fractions, but with much lower peak abundances than AOB, indicating substantially lower isotopic enrichment of AOA than of AOB (Fig. 1h). In the NH_4_Cl treatment, the observed density shift may reflect contributions from both ^13^C and ^15^N incorporation, which were not quantified separately. Together, these SIP results indicate that AOB, rather than AOA, represented the dominant actively growing ammonia oxidizers in both urea- and NH_4_Cl-amended soils.

Phylogenetic analysis of *amoA* genes recovered from total soil DNA at Day 0 and from isotope-labelled higher-density DNA fractions at Days 42 and 63 revealed distinct changes in the community structure of active ammonia oxidizers (Fig. 1i and j). Active AOB in both urea- and NH_4_Cl-amended soils were primarily affiliated with D12, D11, and D8 clusters (Fig. 1i). Notably, D12 cluster was the dominant active AOB cluster in the urea-amended soils, whereas D11 cluster predominated in the NH_4_Cl-amended soils (Fig. 1i). Furthermore, the sequencing profiles showed higher relative abundances of the dominant isotope-labelled AOB D11 and D12 clusters after prolonged incubation (Fig. 1i). In contrast, active AOA in both urea- and NH_4_Cl-amended soils were almost exclusively affiliated with clade NS-α (Fig. 1j).

### Enrichment of an acid-tolerant ammonia-oxidizing bacteria culture

After prolonged incubation, the AOB enrichment culture formed visible granular aggregates rather than a homogeneous planktonic suspension, indicating a strongly aggregated mode of growth (Fig. 2a). Nitrification assays across a range of maintained pH conditions showed that activity was highest at pH 7.5–8.0. Although NO_x_^–^-N production decreased substantially at pH 5.0–6.0, nitrification activity remained detectable even at pH 5.0 (Fig. 2b).

**Figure 2:**
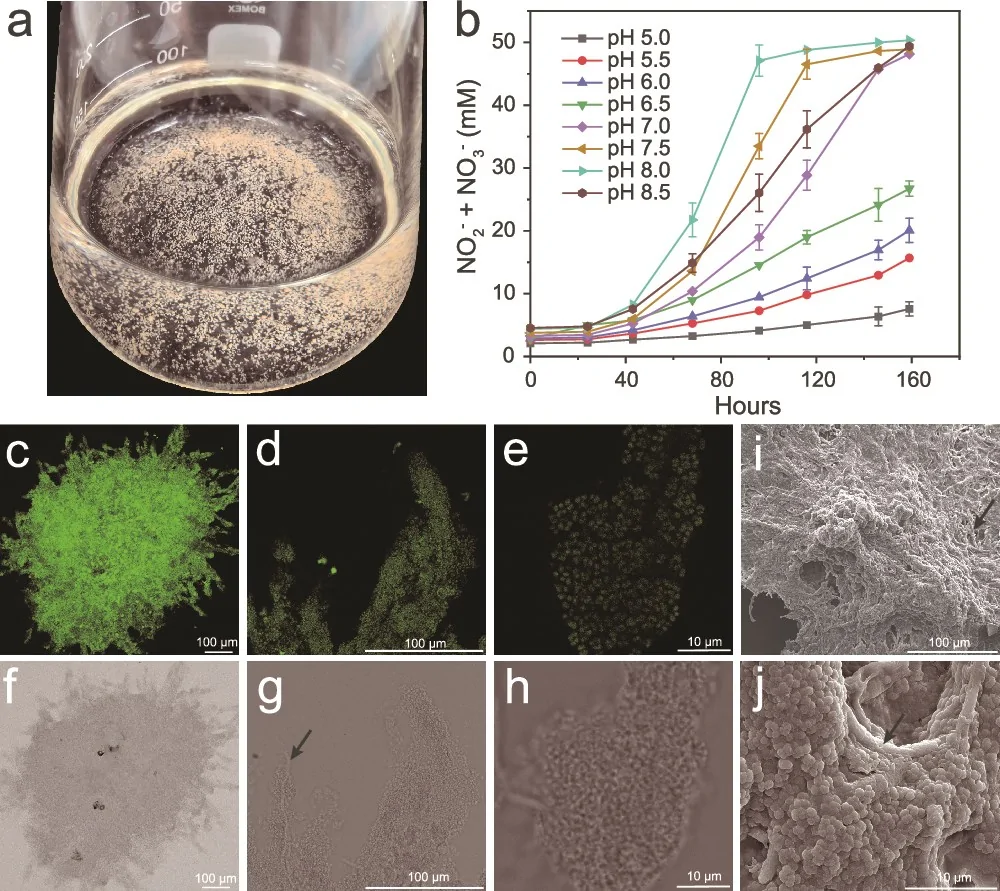
Enrichment and characterization of acid-tolerant AOB. (a) Representative appearance of the long-term enrichment culture maintained at pH 6.0. (b) Nitrification activity of the enrichment culture in medium containing 50 mM NH_4_^+^-N across a pH range of 5.0–8.5. (c–h) FISH and bright-field micrographs of the enrichment culture. FISH was performed using the Cy3-labeled oligonucleotide probe Nsv443. For visualization, the Cy3 fluorescence signal was pseudocolored during image processing. (i, j) Scanning electron micrographs of the enrichment culture at pH 6.0. Arrows in panels g, i, and j indicate elongated biological structures.

FISH revealed spatially clustered cells hybridizing with the *Nitrosospira*-targeted probe within the enrichment culture aggregates (Fig. 2c–e, S3). The enrichment culture aggregates reached up to 1 mm in length, whereas individual cells were ~1 μm in diameter (Fig. 2e). Bright-field images showed corresponding aggregate boundaries and dense internal organization (Fig. 2f–h). SEM images further revealed block-like attached structures, membrane-like coverage, and mesh-like extracellular material (Fig. 2i). Cells were closely packed within these structures, forming compact three-dimensional aggregates (Fig. 2j). Prominent elongated structures were also observed within the cell aggregates, particularly in Fig. 2g, i, and  j.

### Metagenome assembly of acid-tolerant ammonia-oxidizing bacteria

Although both D11 and D12 clusters AOB were active during soil acidification, a high-quality AOB MAG affiliated with D11 cluster was recovered from the long-term enrichment culture. The *amoA* sequence recovered from this MAG clustered within D11 cluster and showed the highest nucleotide identity (98.2%) to SIP-derived AOB-*amoA*-OTU-9 (Fig. S4a), providing a sequence-level link between the enrichment-derived population and an active soil D11 lineage. Therefore, subsequent genomic and transcriptomic analyses focused on this enrichment-derived D11 population as a representative of an active D11 cluster rather than of the entire active AOB community. Phylogenomic analysis placed the enrichment-derived MAG within the genus *Nitrosospira*, where it formed a strongly supported sister lineage to *Nitrosospira* sp. TNL300 (Fig. 3a). This taxonomic placement was further supported by congruent phylogenies based on the *amoA* and 16S rRNA genes (Fig. S4). The enrichment-derived MAG shared 94.9% average nucleotide identity (ANI) and 94.6% average amino acid identity (AAI) with *Nitrosospira* sp. TNL300 (Fig. 3a). Based on its phylogenetic placement and genomic relatedness, this MAG was designated *Nitrosospira* sp. NT. Hereafter, *Nitrosospira* sp. NT refers to this enrichment-derived MAG and its corresponding representative population rather than a pure isolate. The MAG of *Nitrosospira* sp. NT was recovered as a high-quality MAG, with 99.0% completeness and 1.6% contamination. The genome was 3 352 347 bp in size, with a coding density of 0.861, a GC content of 54.0%, and 3134 predicted coding sequences. Circular visualization of the genome assembly was consistent with a high-quality assembly, showing an even distribution of coding sequences across the genome and regular patterns of GC content and GC skew, with no obvious large-scale anomalous regions (Fig. 3b). Together, these features indicate a robust assembly suitable for downstream comparative genomic and metabolic analyses. Phage-associated genes were also identified in the *Nitrosospira* sp. NT MAG. Phylogenetic analysis of a tail sheath protein grouped this sequence with phage proteins affiliated with *Caudoviricetes*/*Caudovirales* (Fig. 3c). In addition, the genome encoded several other phage-related structural proteins, including a tail sheath protein, tail proteins, and a baseplate wedge protein (Fig. S5a–c).

**Figure 3:**
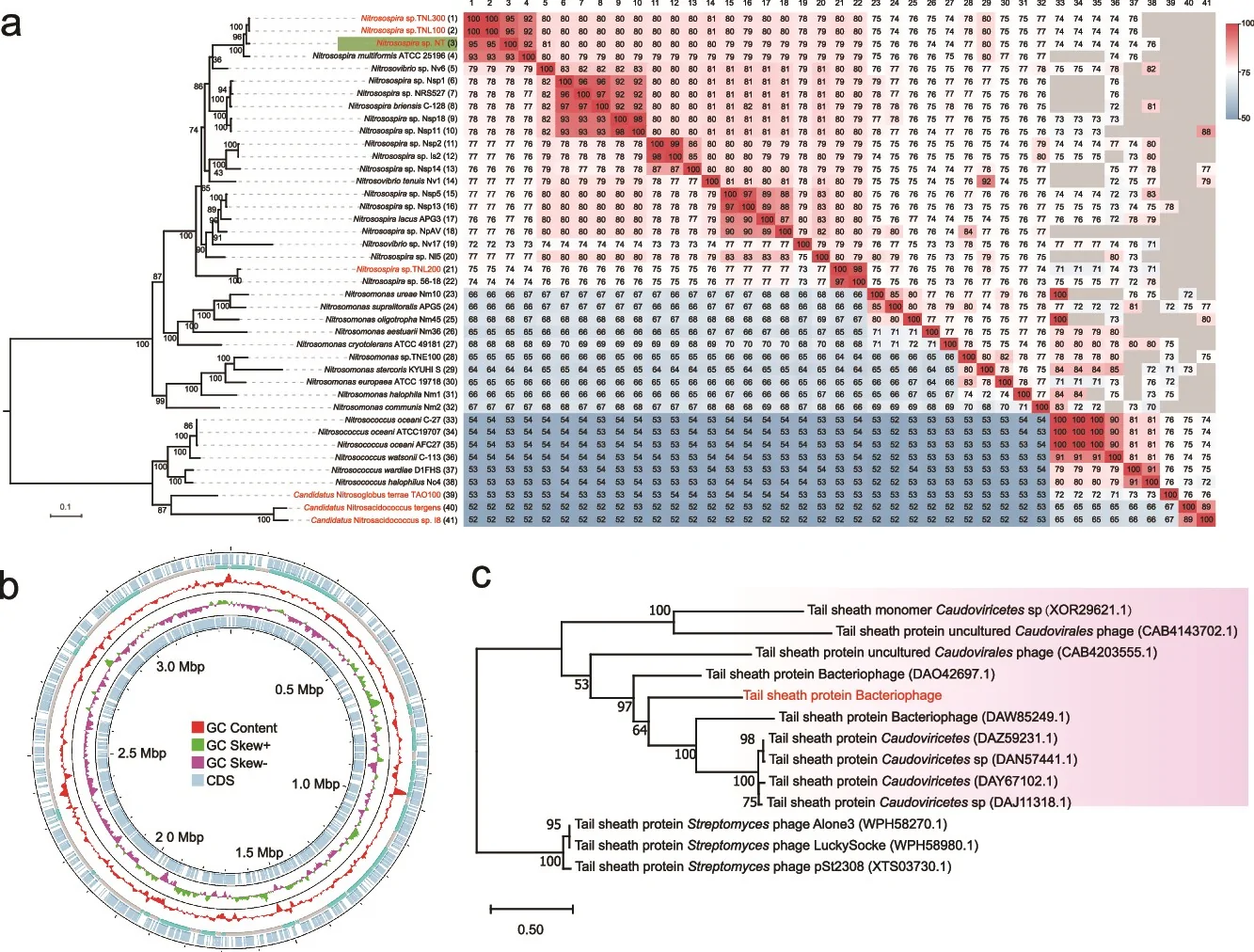
Genomic analysis of the enrichment-derived *Nitrosospira* sp. NT MAG. (a) Phylogenetic placement of *Nitrosospira* sp. NT and comparisons of ANI and AAI with representative AOB genomes. Some AOB representatives included in the phylogenetic tree have been reported to grow or retain ammonia-oxidizing activity at pH ≤ 6.0. AAI values are presented in the lower-left triangle, whereas ANI values are presented in the upper-right triangle, with only values ≥70% shown. Genomic information for the AOB used to construct the phylogenetic tree is provided in Supplementary Table S2. (b) Circular genome map of *Nitrosospira* sp. NT. (c) Phylogenetic tree of a phage-associated tail sheath protein encoded in the *Nitrosospira* sp. NT MAG. Phylogenetic tree was constructed using IQ-TREE v1.6.12 with the best-fit model selected by BIC (WAG+F + I + G4), and branch support was estimated from 1000 bootstrap replicates.

### Aggregation and fungal occurrence in the ammonia-oxidizing bacteria enrichment culture

Under acidic conditions, fungal occurrence coincided with pronounced aggregation of the enrichment culture. When the pH 6.0 stock culture was serially propagated in media adjusted to either pH 6.0 or pH 8.0, the culture maintained at pH 6.0 consistently exhibited abundant dense granular aggregates throughout propagation, whereas the culture transferred to pH 8.0 became progressively more dispersed after several generations, with finer suspended particles and higher turbidity (Fig. 4a and b). Upon plating on PDA, white floccose fungal growth was observed from the pH 6.0 enrichment culture, whereas no visible fungal growth was observed on plates inoculated with the pH 8.0 enrichment culture under the same conditions (Fig. 4c and d). No visible fungal growth was observed on uninoculated negative-control plates. Fungal colonies recovered from the pH 6.0 culture were repeatedly purified, yielding morphologically uniform cottony colonies (Fig. 4e). DNA extraction from the pH 6.0 and pH 8.0 cultures (Fig. 4a and b), followed by fungal DNA quantification and gel electrophoresis, revealed abundant fungal DNA in the pH 6.0 culture, whereas fungal DNA was barely detectable in the pH 8.0 culture (Fig. S6). ITS sequencing and phylogenetic analysis placed the fungal sequences recovered from the enrichment culture and the purified fungal isolate within the genus *Fusarium* (Fig. 4g). SEM imaging revealed tightly interwoven hyphae of the fungal isolate, forming a dense reticulate structure (Fig. 4f), which morphologically resembled the elongated biological structures observed in Fig. 2g, i, and  j. We further compared the nitrification activity of cultures maintained long term at pH 6.0 or pH 8.0 across a range of assay pH values. Both cultures retained nitrification activity across pH 5.0–9.0. Notably, the culture maintained at pH 6.0 exhibited relatively higher activity under acidic conditions, particularly at pH 5.0 (Fig. S7).

**Figure 4:**
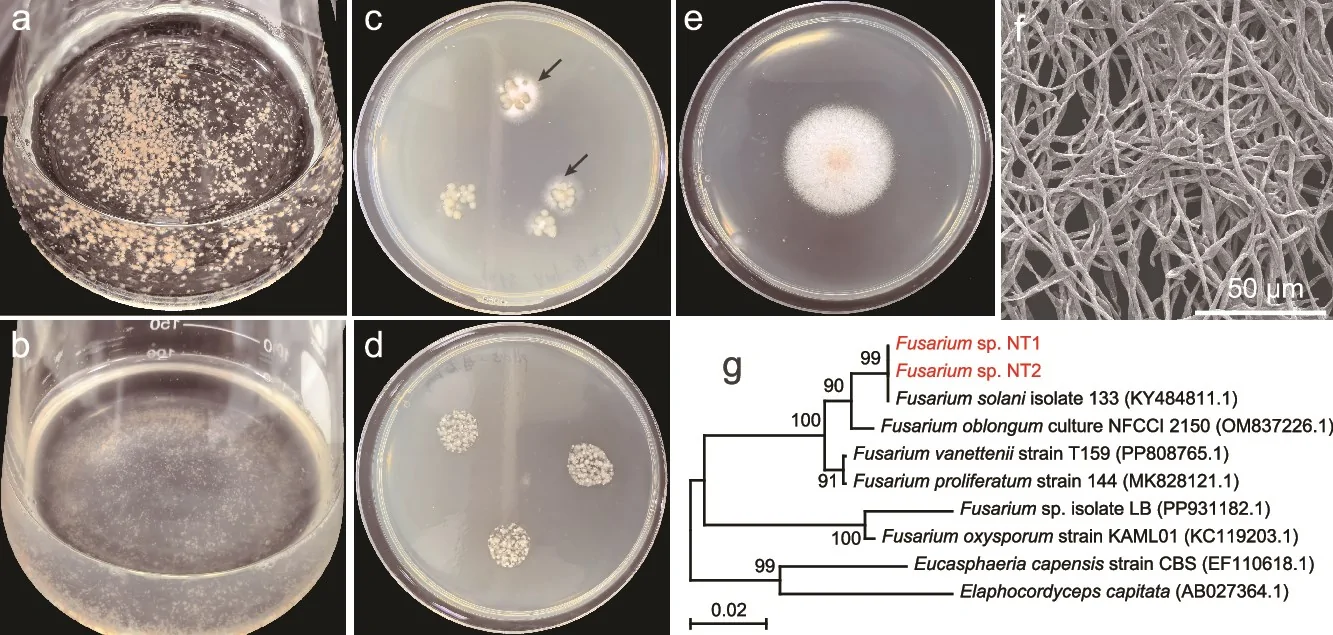
Aggregation and fungal occurrence in the enrichment culture under acidic conditions. (a, b) Representative appearances of the enrichment culture after serial propagation at pH 6.0 and pH 8.0, respectively. (c, d) PDA plates inoculated with cultures grown at pH 6.0 and pH 8.0, respectively. (e) Colony morphology of the fungal isolate obtained from the pH 6.0 enrichment culture on PDA. (f) Scanning electron micrograph of the fungal isolate. (g) Phylogenetic placement of the fungal isolate inferred from ITS1 sequences. *Fusarium* sp. NT1 and *Fusarium* sp. NT2 were identified by sequencing the enrichment culture shown in panel a and the fungal isolate shown in panel e, respectively. Phylogenetic tree was constructed using IQ-TREE v1.6.12 with the best-fit model selected by BIC (TNe + G4), and branch support was estimated from 1000 bootstrap replicates. The image in panel a was obtained from a separate experimental batch from that shown in Fig. 2a.

### Transcriptional traits associated with acid tolerance

Compared with pH 8.0, the enrichment-derived *Nitrosospira* sp. NT population exhibited a pronounced transcriptional response at pH 5.0, with 438 genes significantly upregulated and 256 genes significantly downregulated (Fig. S8, Table S3). Based on these transcriptomic differences, we further analyzed the metabolic characteristics of *Nitrosospira* sp. NT under acidic conditions.

Two-component systems are among the most prevalent mechanisms for environmental sensing and signal transduction in bacteria, generally comprising a sensor histidine kinase (HK) and a cognate response regulator (RR) [38, 39]. The *Nitrosospira* sp. NT MAG harbors multiple *evgS* homologs encoding sensor HKs. Among these, *evgS*-1 was significantly upregulated at pH 5.0, together with the response regulator gene *glrR* (Fig. 5). In addition, multiple genes encoding components of multidrug efflux systems, including *acrA*, *acrB*, *oprM*, *mdtA*, *mexF*, and *vcaM*, were broadly upregulated (Fig. 5), suggesting that these efflux systems may contribute to the acid-responsive transcriptional profile.

**Figure 5:**
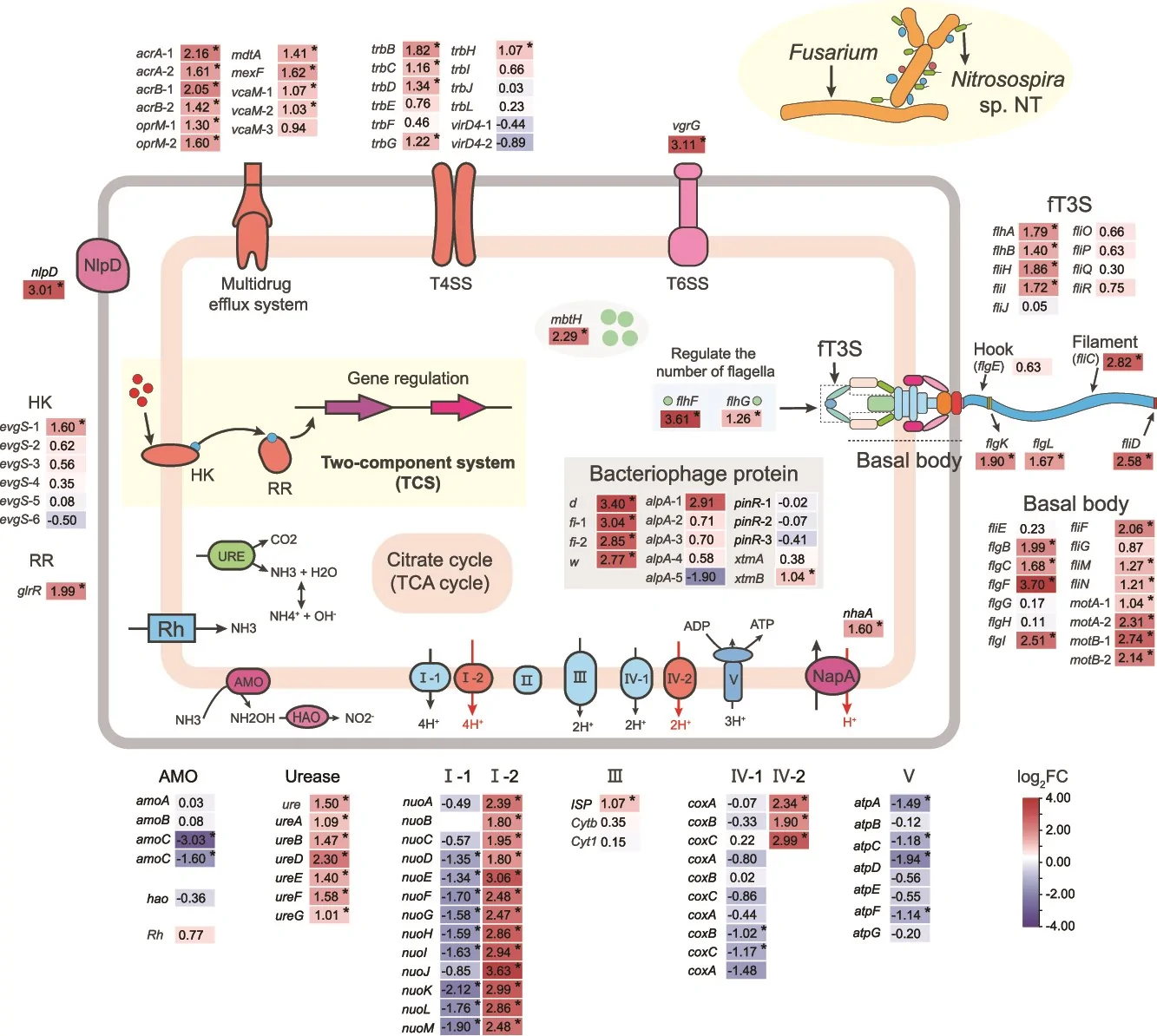
Transcriptional responses of the enrichment-derived *Nitrosospira* sp. NT population to acidic conditions. Transcriptomic changes of *the* enrichment-derived *Nitrosospira* sp. NT population at pH 5.0 were analyzed with pH 8.0 as the reference condition. Data are shown as log_2_(fold change). Asterisks indicate significantly differentially expressed genes (adjusted *P* < .05). I, II, III, IV, V indicate electron transport chain complexes I, II, III, IV, V, respectively. *nhaA* NhaA-type Na^+^/H^+^ antiporter (NhaA); *amoA*–*C* subunits of ammonia monooxygenase (AMO); *hao* hydroxylamine oxidoreductase (HAO); *ure* urease (URE); *nuoA*–*M* subunits of complex I; *ISP*/*Cytb*/*Cyt1* subunits of complex III; *coxA*–*C* subunits of complex IV; *atpA*–*G* subunits of complex V; HK histidine kinase; RR response regulator; *evgS* sensor histidine kinase EvgS; *glrR* response regulator; *nlpD* lipoprotein NlpD; *d* bacteriophage probable baseplate hub protein; *fi* bacteriophage tail sheath protein; *w* bacteriophage baseplate protein W; *alpA* prophage regulatory protein; *pinR* putative DNA-invertase from lambdoid prophage Rac; *xtmA* phage terminase small subunit; *xtmB* phage terminase large subunit; T4SS type IV secretion system; T6SS type VI secretion system; *vgrG* T6SS secreted protein VgrG; fT3S flagellar type III secretion system.

Several genes associated with secretion and surface-related cellular functions also showed altered expression under acidic conditions. At pH 5.0, the T6SS-related gene vgrG was significantly upregulated, together with increased expression of several Trb-family genes associated with the T4SS (Fig. 5). Genes involved in flagellar assembly and secretion were also broadly upregulated, including flhF, flhG, components of the flagellar type III secretion system (fT3S), basal body proteins, and hook- and filament-associated proteins (Fig. 5). In addition, several phage-related structural genes, including those encoding tail sheath, tail, and baseplate proteins, showed increased expression under acidic conditions (Fig. 5). These transcriptional changes identify candidate systems potentially associated with surface-related growth or stress responses under acidic conditions.

*Nitrosospira* sp. NT conserved its ammonia-oxidizing metabolism but reconfigured multiple physiological modules under acidic conditions. The expression of the canonical ammonia oxidation genes *amoA* and *amoB* remained largely stable, and *hao* showed only minor changes in expression, whereas two *amoC* copies were markedly downregulated. These patterns indicate that the core ammonia oxidation module was broadly maintained at pH 5.0, with selective adjustment of specific subunits (Fig. 5). In the respiratory chain, genes associated with complex I-2, complex III, and complex IV-2 were significantly upregulated, whereas those related to complex I-1, complex IV-1, and several ATP synthase subunits tended to be downregulated (Fig. 5). At pH 5.0, the urease gene cluster was coordinately upregulated, whereas transcripts encoding the Rh-type ammonium transporter showed little change, suggesting that the enrichment-derived *Nitrosospira* sp. NT population may preferentially enhance urea hydrolysis to generate NH_3_ locally and thereby mitigate local acidification (Fig. 5). At the same time, upregulation of *nhaA* suggests that Na^+^/H^+^ exchange may contribute to intracellular pH homeostasis (Fig. 5). Together, these transcriptional patterns are consistent with coordinated responses involving acid-base buffering and ion homeostasis under acidic conditions.

## Discussion

### Nitrogen fertilizer-induced acidification selects for acid-tolerant ammonia-oxidizing bacteria

Repeated additions of urea or NH_4_Cl promoted nitrification, which accelerated soil acidification and imposed strong acid stress on ammonia oxidizers, driving soil pH from neutral to acidic conditions. DNA-SIP analysis showed substantially stronger isotopic enrichment of AOB *amoA* genes than of AOA *amoA* genes, indicating that AOB were the principal active ammonia oxidizers during nitrogen fertilizer-induced soil acidification (Fig. 1g and h). Phylogenetic analysis further identified the *Nitrosospira* D11 and D12 clusters as the dominant active AOB (Fig. 1i). This finding is consistent with previous SIP-based evidence demonstrating the functional activity of AOB in other acidic soils. In an acidic forest soil (pH 5.0), ^13^C-DNA-SIP identified *Nitrosospira*-related AOB as active autotrophic ammonia oxidizers [21], while *Nitrosospira* cluster 3-like AOB were shown to dominate bacterial ammonia oxidation in acidic terrace paddy soils [22]. These independent observations support the functional importance of AOB in some acidic soils, although the relative contributions of AOB and AOA are likely to vary with soil properties and environmental conditions. However, because the present study examined only one maize-cultivated black soil, whether acid-tolerant AOB similarly dominate active ammonia oxidation across other acidifying agricultural soils remains to be determined. By contrast, D6 and D7 clusters declined during acidification, consistent with their known association with neutral to alkaline soils [40, 41]. The AOA community also showed acidification-associated selection, with active AOA predominantly shifting toward the clade NS-α, a group previously shown to be widespread and active in acidic soils [42–44]. Nevertheless, the much stronger isotopic enrichment of AOB than AOA indicates that AOB were the dominant active group among the canonical ammonia oxidizers in the maize-cultivated black soil examined here under repeated nitrogen addition. This AOB dominance was likely reinforced by high N inputs, because AOA are generally better adapted to low-ammonium environments and can be inhibited by high ammonium concentrations [12, 45], whereas AOB tend to thrive under elevated ammonium conditions and often dominate nitrification in fertilized agricultural soils [9, 18, 20]. Consistently, previous studies have also shown that archaeal *amoA* mRNA was not fully labeled at a nitrogen addition rate of 100 μg g^−1^ dry soil compared with 15 μg g^−1^ dry soil, suggesting that high N inputs can suppress AOA activity [25].

Comammox *Nitrospira* represent an additional ammonia-oxidizing guild that may contribute to nitrification in agricultural soils, including under acidic conditions [46, 47]. However, the DNA-SIP analyses in the present study specifically targeted the *amoA* genes of canonical AOB and AOA and did not directly quantify comammox *Nitrospira*. Therefore, although the substantially stronger isotope labeling of AOB than AOA indicates that AOB were the predominant active group among the canonical ammonia oxidizers examined here, our data do not exclude a contribution from comammox *Nitrospira* to nitrification. The relative contributions of AOB, AOA, and comammox *Nitrospira* are likely to depend on environmental factors such as soil pH and ammonium availability. Future studies using comammox-specific DNA-SIP or other targeted approaches will be required to determine their contribution during progressive nitrogen fertilizer-induced acidification.

Collectively, these results indicate that repeated nitrogen addition promoted nitrification and soil acidification in the tested black soil while favoring acid-tolerant ammonia-oxidizing lineages. Among the canonical ammonia oxidizers examined, *Nitrosospira*-related AOB, particularly D11 and D12 clusters, represented the dominant active AOB under these experimental conditions. Although both D11 and D12 contributed substantially to active ammonia oxidation in the soil microcosms, the enrichment-derived AOB population recovered for genomic and transcriptomic characterization belonged to D11. Its *amoA* sequence was most closely related to the actively labelled D11 cluster OTU-9 (98.2% nucleotide identity; Fig. S4a). Therefore, the enrichment-derived *Nitrosospira* sp. NT provides a representative model for one active D11 cluster, and the physiological and transcriptional traits described below should not be assumed to represent all D11 cluster populations or the co-occurring D12 cluster AOB.

### Physiological and transcriptional responses associated with acid tolerance

Although the enrichment culture was maintained at pH 6.0, its highest nitrification activity occurred at pH 7.5–8.0. This apparent discrepancy likely reflects a distinction between acid tolerance and pH optimum. The pH 6.0 enrichment condition was used as a selective pressure to favor AOB capable of maintaining activity under moderately acidic conditions, rather than to select for obligately acidophilic organisms. Similar patterns have been reported for AOB inhabiting acidic environments. In particular, *Nitrosospira* strains TNL100, TNL200, and TNL300 isolated from strongly acidic tea-field soils (pH 3.2–5.5) grew over pH 6.0–8.0 but exhibited optimal growth at pH 7.5 [28]. Likewise, the acid-tolerant γ-AOB Ca. Nitrosoglobus terrae TAO100 can persist under highly acidic conditions while growing over a substantially higher pH range [23], and Ca. Nitrosacidococcus tergens RJ19 can oxidize ammonia at pH 2.5 despite exhibiting its highest activity at pH 6.0 [24]. Thus, the ability of the enrichment culture to retain nitrification activity at pH 5.0, while showing maximal activity at pH 7.5–8.0, is consistent with an acid-tolerant rather than obligately acidophilic phenotype.

#### Substrate acquisition and acid buffering

Transcriptomic responses of the enrichment-derived *Nitrosospira* sp. NT population suggest that urea hydrolysis may contribute to ammonia oxidation under acidic conditions. AMO uses NH_3_ rather than NH_4_^+^ as its direct substrate [13, 48]. Therefore, decreasing pH strongly reduces NH_3_ availability by shifting the NH_3_/ NH_4_^+^ equilibrium toward NH_4_^+^ (NH_3_ + H^+^ ↔ NH_4_^+^; pKa = 9.25) [5]. Under such conditions, urea hydrolysis can partially alleviate substrate limitation by releasing NH_3_ locally ((NH_2_)_2_CO + H_2_O → 2NH_3_ + CO_2_), thereby supporting AOB activity even at low pH [49]. In addition, NH_3_ released from urea hydrolysis can generate OH^−^ during conversion to NH_4_^+^ (NH_3_ + H_2_O ↔ NH_4_^+^ + OH^−^), potentially contributing to intracellular proton neutralization and pH buffering [5, 50]. Ureolysis may represent a recurrent strategy among acid-tolerant AOB rather than a feature specific to *Nitrosospira* sp. NT. Urease systems and urea utilization have also been reported in the acid-tolerant γ-AOB Ca. Nitrosoglobus terrae TAO100 and Ca. Nitrosacidococcus tergens RJ19, as well as in *Nitrosospira* strains TNL100, TNL200, and TNL300 isolated from acidic tea-field soils [23, 24, 28]. Thus, urea hydrolysis may provide a broadly shared advantage under acidic conditions by facilitating ammonia acquisition and potentially contributing to local pH buffering. In *Nitrosospira* sp. NT, the coordinated increase in urease-associated transcripts at pH 5.0 further indicates that this shared metabolic capacity is transcriptionally responsive to acidic conditions (Fig. 5). This interpretation is also supported by soil microcosm results. In these experiments, urea-amended soils maintained higher pH than NH_4_Cl-amended soils, despite comparable or greater NO_x_^–^-N accumulation (Fig. 1a and b). Moreover, the pH increase in soils amended with both urea and acetylene suggests that urea hydrolysis can occur independently of ammonia oxidation, potentially leading to localized alkalinization in soil microsites. Therefore, urea addition may support acid-tolerant AOB by increasing NH_3_ availability and locally elevating extracellular pH, thereby alleviating acidification stress.

#### Intracellular pH and envelope homeostasis

In addition to substrate acquisition, *Nitrosospira* sp. NT appears to maintain ammonia-oxidizing activity under acidic stress by coordinating energy metabolism, ion transport, and envelope stress responses (Fig. 5). The transcriptional maintenance of the core ammonia-oxidation machinery suggests that ammonia oxidation remained operational at low pH, whereas selective changes in AMO-associated genes may reflect stress-related adjustments of this pathway [51]. Such remodeling may allow *Nitrosospira* sp. NT to sustain ammonia oxidation while adjusting its energy metabolism to acidic conditions.

The respiratory electron transport chain also appeared to be reconfigured under acid stress. The preferential induction of specific respiratory complexes, along with reduced expression of several ATP synthase-related genes, may reflect a shift toward enhanced proton extrusion and rebalancing of the proton motive force. Similar strategies have been reported in neutrophilic bacteria such as *Escherichia coli*, where acid stress induces proton-exporting respiratory chain components while reducing ATP synthase activity to help maintain intracellular pH homeostasis [52]. In addition, AOA have been proposed to adapt to acidic environments in part through V-type ATPases, which may help export cytoplasmic protons and thereby support pH homeostasis under low-pH conditions [53]. By analogy, respiratory remodeling in *Nitrosospira* sp. NT, particularly the upregulation of complex I-2 and complex IV-2, may help maintain acid-base homeostasis across the cell membrane by promoting proton translocation and proton motive force rebalancing. Maintenance of proton and ion homeostasis also appears to be a common theme among acid-tolerant AOB. Cation- and proton-transport systems have been implicated in the acid adaptation of *Ca*. Nitrosoglobus terrae TAO100 and *Ca*. Nitrosacidococcus tergens RJ19 [23, 24]. In *Nitrosospira* sp. NT, the increased expression of the Na^+^/H^+^ antiporter gene *nhaA*, together with the observed changes in respiratory-chain components, is therefore consistent with a broader strategy of adjusting proton transport and energy metabolism to maintain intracellular pH under acidic conditions [23, 24].

Envelope stress sensing and defense likely represent an additional layer of acid adaptation. The induction of two-component regulatory systems suggests enhanced environmental sensing and signal transduction under low-pH stress. In particular, EvgS-like HKs and response regulator GlrR may participate in acid-responsive regulation, as similar systems have been linked to acid resistance and cell envelope integrity in other bacteria [54–56]. In parallel, activation of lipoprotein- and multidrug efflux-related functions suggests enhanced membrane maintenance, detoxification, and overall envelope protection [57–60]. Together, these responses may contribute to intracellular pH homeostasis and cell-envelope protection under acidic conditions.

#### Aggregate formation and potential microbial associations

Cellular aggregation may provide protection against environmental stress by modifying diffusion and creating microscale conditions that differ from the surrounding bulk phase in pH, nutrient availability, and toxic metabolite concentrations [61–64]. Aggregation has also been observed in other acid-tolerant or acid-associated AOB, suggesting that aggregation-associated protection may represent a recurrent response to acidic environments. However, the mechanisms underlying aggregate formation may differ among AOB lineages. Ca. Nitrosoglobus terrae TAO100 survives highly acidic conditions through cell aggregate formation despite lacking identifiable flagellar genes [23]. In contrast, the acid-associated *Nitrosospira* strains TNL100, TNL200, and TNL300 isolated from tea-field soils all form aggregates and encode most components of the bacterial flagellar system, together with putative EPS- and LPS-related functions [28].

In *Nitrosospira* sp. NT, several flagellar assembly and regulatory genes were transcriptionally responsive to acidic conditions. The occurrence of flagellar machinery in both *Nitrosospira* sp. NT and the acid-associated TNL *Nitrosospira* strains suggests that flagellar functions may represent a recurrent feature of these *Nitrosospira* lineages, potentially contributing to motility, surface sensing, or attachment [28]. However, the present data do not establish a direct role for flagella in aggregate formation or acid tolerance in *Nitrosospira* sp. NT, and this possibility remains to be experimentally tested. The co-occurrence of fungi and pronounced aggregation under acidic conditions further suggests a potential association between fungal occurrence and the aggregated phenotype of the enrichment culture. However, the present data do not establish whether fungi directly contribute to aggregate formation or acid tolerance. Thus, fungal involvement should currently be regarded as a potential ecological association rather than a demonstrated mechanism.

Acid-responsive changes were also observed in genes associated with T4SS- and T6SS-related systems. These transcriptional responses identify candidate systems potentially involved in cell-surface or intermicrobial responses, but do not provide direct evidence for molecular interactions with fungi or neighboring bacteria. Several phage-related structural genes also exhibited altered transcription under acidic conditions. Because contractile phage tails and T6SS share homologous structural components [65, 66], the phage-related structural genes identified in the *Nitrosospira* sp. NT genome are considered potential T6SS-associated components. Notably, these phage-related genes are located adjacent to the gene encoding VgrG, a core structural component of T6SS, suggesting a possible genomic association with a T6SS-related system (Table S3). However, whether these genes function as components of T6SS or other phage-like structures remains to be experimentally validated.

Overall, comparison with previously characterized acid-tolerant AOB suggests that ureolysis, proton/ion homeostasis, and cellular aggregation represent recurrent or convergent strategies for persistence under acidic conditions. The distinctive feature of *Nitrosospira* sp. NT therefore does not appear to be a single unique acid-tolerance mechanism, but rather the coordinated acid-responsive regulation of these processes, together with flagellar and envelope-associated functions.

### Ecological and agricultural implications

Northeast China’s black soils support ~20% of the national maize-growing area and over 30% of total maize production, underscoring their agricultural importance [30]. In this maize-based upland black soil, acid-tolerant AOB sustained soil nitrification under fertilization-induced acidification, indicating an important role in nitrogen cycling under the conditions tested. Given the importance of NO_3_^−^ as an inorganic nitrogen source for maize [67], continued nitrification under acidifying conditions may influence nitrogen availability in this system. However, the persistence of nitrification as soil pH declines may also prolong nitrification-associated proton production, indicating a potential trade-off between maintaining oxidative nitrogen transformation and further soil acidification [1]. The suppression of both nitrification and soil acidification when ammonia oxidation was inhibited in our microcosms further supports this close coupling (Fig. 1a and b). From a nitrogen-management perspective, these findings suggest that avoiding excessive N inputs and improving the synchronization of fertilizer application with crop N demand may reduce the amount of NH_4_^+^ available for sustained nitrification and thereby help slow soil acidification [68–70]. Approaches such as optimized N application rates, split fertilization, and, where appropriate, nitrification-inhibiting or other enhanced-efficiency fertilizer strategies may therefore be useful for reducing nitrification-associated acidification [68]. The stronger acidification observed following NH_4_Cl than urea addition further suggests that fertilizer N form may influence the magnitude of acidification [69, 70], although this effect requires validation under field conditions. Previous SIP studies have also demonstrated functionally active *Nitrosospira*-related AOB in acidic forest and paddy soils [21, 22], suggesting that the contribution of AOB to nitrification under acidic conditions is not restricted to the soil examined here. However, because only one agricultural soil was investigated in the present study, the prevalence and relative importance of acid-tolerant AOB across acidifying agricultural soils remain to be established. Likewise, the potential effectiveness of the nitrogen-management strategies discussed above should be evaluated across different soils, crops, and fertilization regimes under field conditions. Furthermore, the co-occurrence of fungi and pronounced aggregation in the enrichment culture under acidic conditions highlights a potential role for microbial associations that warrants further investigation. Together, these findings highlight the ecological significance of acid-tolerant AOB in the maize-cultivated black soil examined here and provide a basis for evaluating their roles in other acidifying agricultural soils and for developing nitrogen-management practices that balance crop N supply with the mitigation of soil acidification.

## Supplementary Material

Supplementary_material_ycag285

## Data Availability

The raw sequencing data generated in this study have been deposited in the NCBI Sequence Read Archive (SRA). The metagenomic raw reads are available under accession number SRR38269913, the metatranscriptomic raw reads under accession numbers SRR38269907–SRR38269912, and the *amoA* amplicon sequencing raw reads under accession numbers SRR38269353–SRR38269365. The enrichment-derived *Nitrosospira* sp. NT MAG has been deposited in NCBI under accession number JCAHZJ000000000. Representative *amoA* gene sequences obtained in this study have been deposited in GenBank under accession numbers PZ378855–PZ378879. Relevant sample metadata were deposited together with the corresponding sequencing datasets in NCBI. The gene count table used for the metatranscriptomic analysis is provided in Supplementary Table S3.
