# Supplementary material for: Ammonia-oxidizing bacteria dominate active ammonia oxidation during nitrogen fertilizer-induced acidification of a maize-cultivated black soil
Source: ISME Commun. 2026 Oct 14;6(1):ycag285. doi: 10.1093/ismeco/ycag285 (PMC13723767; doi:10.1093/ismeco/ycag285)
Supplement: Supplementary file 1 [file supplementary_material_ycag285.zip › Supplementary Information.docx]

**Supplementary Online Material For**

Ammonia-oxidizing bacteria dominate active ammonia oxidation during nitrogen fertilizer-induced acidification of a maize-cultivated black soil

**This file includes:**

Supplementary methods

Figure S1–S8

Table S1–S3

References

**Supplementary methods**

**Soil characteristics**

Soil pH was measured in a soil–water suspension prepared at a 1:2.5 (w/w) ratio using an FE28-Standard pH meter (Mettler Toledo Instrument, Shanghai, China). The contents of soil organic matter (SOM) and soil organic carbon (SOC) were determined with an HH-SA analyzer (Ronghua Instrument Manufacturing, Changzhou, China). Total nitrogen (TN) was measured using an ATN-300 instrument (CANY, Shanghai, China), whereas hydrolyzable nitrogen (HN) was analyzed with an LRH-250 instrument (YIHENG, Shanghai, China). Total phosphorus (TP) was quantified with a FIexA-200 spectrophotometer (ALPHA, Suzhou, China), and available phosphorus (AP) was determined using a UV 8000 spectrophotometer (METASH, Shanghai, China). Total potassium (TK) and available potassium (AK) were both measured with an FP6431 analyzer (INESA, Shanghai, China).

**Determination of NH_4_^+^, NO_2_^−^ and NO_3_^−^**

The concentrations of NH_4_^+^, NO_2_^−^ and NO_3_^−^ were determined in 1 mL culture samples after centrifugation at 14,000 rpm for 2 min. The resulting supernatant was immediately diluted with H_2_O, and the concentrations of the three ions were subsequently determined using an MQX200 microplate reader (BioTek, Vermont, USA) [1].

**pH-gradient nitrification assay**

The long-term pH 6.0 enrichment culture was used as the inoculum for the pH-gradient nitrification assay. The enrichment culture was inoculated into AOB media adjusted to pH 5.0, 5.5, 6.0, 6.5, 7.0, 7.5, 8.0, or 8.5, with three biological replicates for each pH treatment. The medium contained 50 mM NH_4_^+^-N, and MES and HEPES were added as buffering agents. Cultures were incubated aerobically at 28 °C in the dark with shaking at 180 rpm. During incubation, medium pH was measured daily and readjusted to the initially designated pH value using 1 M NaOH or 1 M HCl as required. Concentrations of NH_4_^+^, NO_2_^−^, and NO_3_^−^ were also measured daily. Nitrification activity was evaluated based on the accumulation of NO_x_^−^-N, defined as the sum of NO_2_^−^-N and NO_3_^−^-N.

For the comparison shown in Fig. S7, enrichment cultures maintained at pH 6.0 or pH 8.0 were used separately as inocula and transferred into AOB media adjusted to pH 5.0, 6.0, 7.0, 8.0, or 9.0. The subsequent incubation conditions, daily pH adjustment, and measurements of NH_4_^+^, NO_2_^−^, and NO_3_^−^ followed the procedures described above. Relative nitrification activity was calculated from NO_x_^−^-N accumulation, using the condition showing the highest activity for each inoculum as the reference.

**Sequencing of *amoA* genes**

For *amoA* sequencing of isotope-labelled higher-density DNA fractions, one biological replicate (n = 1) was selected from the three independently fractionated SIP replicates for each corresponding treatment and sampling time. Archaeal *amoA* genes were amplified by PCR using the primer set Arch-amoAF/Arch-amoAR [2], whereas bacterial *amoA* genes were amplified using the primer set amoA1F/amoA2R [3]. The PCR products were purified via 2% agarose gel electrophoresis and were sequenced on an Illumina HiSeq platform at Sangon Biotech Co., Ltd. (Shanghai, China). The raw FASTQ files were demultiplexed using an in-house Perl script and quality-filtered with Fastp version 0.19.6 [4] with recommended parameters. Sequence merging was conducted using FLASH version 1.2.7 [5]. The resulting sequences were clustered into operational taxonomic units (OTUs) at a 97% sequence similarity level using UPARSE 7.1 [6]. Taxonomic assignments for each representative OTU sequence were performed using the RDP Classifier version 2.2 [7] against the NCBI nucleotide database.

**Fungal isolation and purification**

Fungi were isolated from enrichment cultures maintained at pH 6.0 and pH 8.0. Aliquots (10 μL) of each liquid enrichment culture were inoculated onto potato dextrose agar (PDA; potato infusion powder, 6 g L^−1^; glucose, 20 g L^−1^; agar, 20 g L^−1^) supplemented with ampicillin (150 μg mL^−1^) to suppress bacterial growth. Uninoculated PDA plates supplemented with the same concentration of ampicillin were incubated in parallel as negative controls. All plates were incubated statically at 28 °C in the dark for 2 days. Hyphal tips from the margins of fungal colonies recovered from the pH 6.0 enrichment culture were transferred into potato dextrose water (PDW; potato infusion powder, 6 g L^−1^; glucose, 20 g L^−1^) for propagation and subsequently replated onto PDA. This isolation and purification procedure was repeated several times to obtain purified fungal colonies.

**Quantification of fungal ITS genes**

The abundance of fungal ITS genes was quantified by quantitative PCR using the primer set ITS1F/ITS4R [8]. The amplification efficiency was 86.2%, with an *R*² value of 0.947. Detailed qPCR conditions are provided in Supplementary Table S1.

**ITS Sequencing**

The fungal ITS region in the AOB enrichment cultures was amplified using the primer pair ITS1F (5′-CTTGGTCATTTAGAGGAAGTAA-3′) and ITS4R (5′-TCCTCCGCTTATTGATATGC-3′) [8]. PCR products were verified by 2% agarose gel electrophoresis. Sequencing libraries were prepared using the SMRTbell® Express Template Prep Kit 2.0 (Pacific Biosciences, Menlo Park, CA, USA) and sequenced on the PacBio Sequel II System by Majorbio Bio-Pharm Technology Co., Ltd. (Shanghai, China). Raw PacBio subreads were processed with SMRT Link v8.0 to generate circular consensus sequences (CCSs) using the parameters minFullPass = 3 and minPredictedAccuracy = 0.99. Quality-filtered sequences were denoised using DADA2 [9] within QIIME 2 [10] under default settings to obtain ASVs. Taxonomic classification of fungal ITS ASVs was performed using a UNITE v9.0-based fungal ITS classifier in QIIME 2 [11].

For purified fungal colonies isolated on PDA plates, the fungal ITS region was amplified using the primer pair ITS1 (5′-TCCGTAGGTGAACCTGCGG-3′) and ITS4 (5′-TCCTCCGCTTATTGATATGC-3′) [12]. PCR products were checked by 2% agarose gel electrophoresis and then subjected to Sanger sequencing by Tsingke Biotechnology Co., Ltd (Beijing, China). The obtained sequences were assembled using ContigExpress, and taxonomic identification was performed by BLASTN comparison against the NCBI nucleotide database.

**Metagenomic sequencing and analysis**

DNA extraction was performed using the Fast DNA SPIN Kit (MP Biomedicals, Cleveland, OH, USA) according to the manufacturer's instructions. The quantity and purity of DNA were assessed using a NanoDrop 2000 (Thermo Scientific, Wilmington, DE, USA) and examined via 1% agarose gel electrophoresis. DNA libraries were prepared using the TruSeq™ DNA Sample Prep Kit (Illumina, San Diego, CA, USA) following the manufacturer's instructions. Sequencing was carried out on an Illumina HiSeq platform at Majorbio Bio-Pharm Technology Co., Ltd. (Shanghai, China). Paired-end Illumina reads were assembled de novo using MEGAHIT v1.2.9 [13] with the parameter --kmin-1pass --mem-flag 2. The resulting contigs were binned into metagenome-assembled genomes (MAGs) using MaxBin v2.2.7 [14] with default settings. MAG quality, including completeness and contamination, was evaluated with CheckM2 v1.1.0 [15]. Taxonomic assignment was performed using GTDB-Tk v2.4.0 [16] with the option --skip_ani_screen, and functional annotation was carried out using KOfamScan v1.3.0 [17] against the KEGG database with -E 1e-5.

**Metatranscriptomic sequencing and analysis**

Total RNA was extracted using the RNeasy^®^ PowerSoil^®^ Total RNA Kit (Qiagen, Valencia, CA) according to the manufacturer's protocol. The purity and concentration of the RNA were assessed using a NanoDrop 2000 (Thermo Scientific, Wilmington, DE, USA), and the integrity of RNA was assessed through 1% agarose gel electrophoresis. RNA Quality Number values were determined using an Agilent 5300 Bioanalyzer (Agilent Technologies, Santa Clara, CA, USA). RNA libraries were constructed with the TruSeq™ RNA Sample Prep Kit (Illumina, San Diego, CA, USA) according to the manufacturer's instructions. Sequencing was performed on an Illumina HiSeq platform at Majorbio Bio-Pharm Technology Co., Ltd. (Shanghai, China). Raw reads were quality-filtered using fastp v0.23.4 [4] with default settings. After quality filtering, rRNA reads were identified and removed using SortMeRNA v7.0 against the SILVA SSU (16S/18S) and SILVA LSU (23S/28S) rRNA databases [18, 19]. The remaining non-rRNA reads were used for downstream transcriptomic analysis. The proportion of rRNA reads removed during computational filtering was calculated relative to the total number of clean reads. The resulting non-rRNA reads from each sample were mapped individually to the corresponding metagenome-assembled genomes (MAGs). Transcript abundance was quantified with Salmon v1.10.3 [20] using the parameters -l A --validateMappings --seqBias --gcBias, and gene expression levels were reported as transcripts per million (TPM) [21]. RNA-seq mapping rates for *Nitrosospira* sp. NT were calculated as the proportion of reads assigned to the *Nitrosospira* sp. NT MAG relative to the total number of non-rRNA reads. A coding sequence (CDS) was considered detected within a treatment when it had a non-zero raw read count in at least two of the three biological replicates. Across the six metatranscriptomic libraries, 76.25–89.62% of clean reads were identified as rRNA and removed during computational filtering. Of the remaining non-rRNA reads, 4.42–6.93% were assigned to the *Nitrosospira* sp. NT MAG. Based on the criterion of a non-zero raw read count in at least two of three biological replicates, 3,080 and 3,071 CDSs were detected under pH 5.0 and pH 8.0, respectively.

**Fluorescence in situ hybridization (FISH)**

FISH was performed as described previously with minor modifications [22]. Briefly, 2 mL of culture was centrifuged at 8000 rpm for 2 min, and the collected cells were fixed in 4% paraformaldehyde at 4 °C for 8 h. Fixed cells were washed three times with 0.1 M PBS (pH 7.0), air-dried, and dehydrated through a graded ethanol series (50%, 70%, 90% and 100%) for 5 min each at room temperature. The oligonucleotide probe Nsv443 (5′-CCGTGACCGTTTCGTTCCG-3′) [23], labelled with Cy3, was used for hybridization. Samples were incubated in hybridization buffer containing 20 mM Tris-HCl (pH 7.2), 0.9 M NaCl, 30% formamide and 0.01% (w/v) SDS with the probe at a final concentration of 25 ng μL^−1^ at 46 °C for 2 h. Following hybridization, samples were washed in buffer containing 108 mM NaCl, 20 mM Tris-HCl (pH 7.5), 5 mM EDTA and 0.01% (w/v) SDS at 48 °C for 30 min, briefly rinsed in cold ddH_2_O, and air-dried. Samples were then counterstained with DAPI to visualize total DNA-containing cells, followed by a brief rinse with ddH_2_O. Coverslips were mounted on glass slides and sealed with transparent nail polish. Fluorescence signals were observed using a Stellaris 5 confocal laser scanning microscope (Leica Microsystems, Wetzlar, Germany) equipped with a 100× oil-immersion objective. The Cy3 and DAPI fluorescence channels were acquired separately, and the Cy3 fluorescence channel was displayed in green pseudocolor during image processing for visualization.

**Scanning electron microscopy (SEM)**

For SEM analysis, 2 mL of culture was fixed by adding 100 μL of 50% glutaraldehyde to a final concentration of 2.5% and incubating the mixture at 4 °C for 8 h. Samples were then centrifuged at 6000 rpm for 2 min, and the pellets were washed three times with 0.1 M PBS (pH 7.0). After air drying, the material was dehydrated through a graded ethanol series (30%, 50%, 70%, 90% and 100%) for 10 min each at room temperature, freeze-dried, and sputter-coated with gold. Sample morphology was then examined using an electron microscope (Quanta 400FEG, FEI, OR, USA).

**Phylogenetic analysis**

Reference genome sequences of AOB were collected from the NCBI databases. Maximum-likelihood phylogenetic trees were constructed with IQ-TREE [24] using the best-fit model selected by ModelFinder. Node support was evaluated by 1000 ultrafast bootstrap replicates and 1000 SH-aLRT tests (-m MFP -bb 1000 -alrt 1000 -nt AUTO). Tree visualization and annotation were performed in iTOL [25].

**Average nucleotide identity (ANI) and average amino‐acid identity (AAI) analyses**

In order to evaluate genome-level similarity among bacterial genomes, we calculated the ANI using FastANI v1.33 [26]. All genome assemblies used in this analysis were provided in FASTA format and checked for quality, completeness, and contamination prior to ANI computation. An all-vs-all pairwise ANI comparison was conducted using the --ql and --rl options.

Pairwise AAI between genomes was computed by initially extracting each proteome from its respective FASTA file, using the filename stem as the genome identifier, and establishing a DIAMOND database for each with Diamond v2.1.4 [27]. Subsequently, reciprocal DIAMOND BLASTP searches were then conducted for every pair of genomes (using an e-value of 1×10⁻^5^ and considering the top hit per query). The tabular output was then parsed to retain only the query ID, subject ID, and percent identity, enabling the identification of reciprocal best hits (RBHs). AAI was determined as the average percent identity across all RBH pairs. Finally, these AAI values were organized into a symmetrical matrix and exported as a CSV file for subsequent analyses.

**Figure S1** Changes in soil NH_4_^+^-N concentration during 42- and 63-day stable-isotope probing incubations. The day-0 soil was treated as the common pretreatment baseline and was not included in the two-way ANOVA; asterisks indicate significant differences from the day-0 baseline based on Dunnett’s test (*P* < 0.05).

**Figure S2** Relative abundance of ammonia-oxidizing bacterial (AOB) (**a**) and archaeal (AOA) (**b**) groups in soils under different treatments based on *amoA* amplicon sequencing.

**Figure S3** FISH and DAPI visualization of *Nitrosospira*-related cells within enrichment culture aggregates. (**a**) FISH signals from cells hybridizing with the Cy3-labeled *Nitrosospira*-targeted probe Nsv443, displayed in green pseudocolor. (**b**) DAPI staining of total DNA-containing cells (blue). (**c**) Overlay of the Nsv443-Cy3 and DAPI fluorescence channels. (**d**) Bright-field image of the same field of view. Scale bars, 10 μm.

**Figure S4** Phylogenetic placement of the *amoA* gene (**a**) and **16S rRNA** gene (**b**) recovered from the *Nitrosospira* sp. NT MAG. The *amoA* gene phylogenetic tree was constructed using the shared *amoA* amplicon region of the enrichment-derived MAG, AOB OTUs recovered from the soil SIP experiment, and representative reference sequences. Pairwise nucleotide identities between the MAG-derived *amoA* sequence and SIP-derived AOB OTUs are indicated next to the OTU labels. Relative abundances of these OTUs in the initial soil and isotope-labelled higher-density DNA fractions are shown in Fig. 1i. Phylogenetic trees were constructed with IQ-TREE v1.6.12 using the best-fit models selected by BIC (HKY+F+I+G4 for *amoA* gene and TN+F+I+G4 for **16S rRNA**); branch support was estimated from 1,000 bootstrap replicates.

**Figure S5** Phylogenetic relationships of phage gene-encoded proteins in the genome of *Nitrosospira* sp. NT. (**a**) Phylogenetic analysis of the tail sheath protein. (**b**) Phylogenetic analysis of the tail protein. (**c**) Phylogenetic analysis of the baseplate protein. Phylogenetic trees were constructed with IQ-TREE v1.6.12 using the best-fit models selected by BIC (LG+G4 for tail sheath protein, WAG+I+G4 for **tail protein, and** LG+I+G4 for baseplate wedge protein); branch support was estimated from 1,000 bootstrap replicates.

**Figure S6** Fungal abundance in enrichment cultures at pH 6.0 and pH 8.0. (**a**) Changes in *ITS1* gene abundance under the two pH conditions. (**b**) Agarose gel electrophoresis of *ITS1* gene PCR products.

**Figure S7** Nitrification activity of enrichment inocula obtained at pH 6.0 and pH 8.0 across different pH conditions. (**a**) **NO_x_⁻-N** concentrations in the enrichment cultures. (**b**) Relative nitrification activity under different pH conditions. After inoculation into media with pH values ranging from 5.0 to 9.0, the inoculum derived from the pH 6.0 enrichment showed the highest nitrification activity at pH 7.0; relative nitrification activity at the other pH values was calculated using the **NO_x_⁻-N** concentration after 72 h in the pH 7.0 medium as the reference. The inoculum derived from the pH 8.0 enrichment showed the highest nitrification activity at pH 8.0; relative nitrification activity at the other pH values was calculated using the **NO_x_⁻-N** concentration after 72 h in the pH 8.0 medium as the reference. Medium pH was monitored daily and readjusted to the designated pH values as required.

**Figure S8** Volcano plot of differentially expressed genes in the enrichment culture under pH 5.0 and pH 8.0 conditions. Statistical analyses were performed using the DESeq2 package in RStudio with specified parameters (|log2FC| > 1, adjusted *P* < 0.05). Three replicates (N = 3) were used for the transcriptomic analyses.

**Table S1** Primers and conditions used in this study.

| Primer Name | Primer sequence (5'-3') | Target gene | Thermal Profile | Molecular analysis | Reference |
| --- | --- | --- | --- | --- | --- |
| Arch-amoAF | STA ATG GTC TGG CTT AGA CG | AOA *amoA* gene | 95°C, 3.0min; 35 cycles × (95°C, 30s; 55°C, 30s; 72°C, 45s with plate read); Melt curve 65.0°C to 95.0°C, increment 0.5°C, 0:05+ plate read | Real-Time PCR sequencing | [2] |
| Arch-amoAR | GCG GCC ATC CAT CTG TAT GT |  |  |  |  |
| amoA-1F | GGG GTT TCT ACT GGT GGT | AOB *amoA* gene | 95°C, 3.0min; 35 cycles × (95°C, 30s; 55°C, 30s; 72°C, 45s with plate read); Melt curve 65.0°C to 95.0°C, increment 0.5°C, 0:05+ plate read | Real-Time PCR sequencing | [3] |
| amoA-2R | CCC CTC KGS AAA GCC TTC TTC |  |  |  |  |
| ITS1F | CTTGGTCATTTAGAGGAAGTAA | ITS | 95°C, 3.0min; 35 cycles × (95°C, 30s; 55°C, 30s; 72°C, 45s) | Real-Time PCR sequencing | [8] |
| ITS4R | TCCTCCGCTTATTGATATGC |  |  |  |  |
| ITS1 | TCCGTAGGTGAACCTGCGG | ITS | 95°C, 3.0min; 35 cycles × (95°C, 30s; 55°C, 30s; 72°C, 45s) | sequencing | [12] |
| ITS4 | TCCTCCGCTTATTGATATGC |  |  |  |  |

**Table S2** The accession numbers and features of all known genomes utilized in this study.

This table is provided as an Excel file because it exceeds one page.

**Table S3** KEGG annotations and metatranscriptomic read counts of coding sequences (CDSs) in the *Nitrosospira* sp. NT MAG.

This table is provided as an Excel file because it exceeds one page.

**References**

1. Hernández-López J, Vargas-Albores F. A microplate technique to quantify nutrients (NO_2_^−^, NO_3_^−^, NH_4_^+^ and PO_4_^3−^) in seawater. *Aquaculture Research* 2003;**34**:1201-04. <https://doi.org/10.1046/j.1365-2109.2003.00928.x>

2. Francis CA, Roberts KJ, Beman JM, Santoro AE, Oakley BB. Ubiquity and diversity of ammonia-oxidizing archaea in water columns and sediments of the ocean. *Proc Natl Acad Sci U S A* 2005;**102**:14683-88. <https://doi.org/10.1073/pnas.0506625102>

3. Rotthauwe JH, Witzel KP, Liesack W. The ammonia monooxygenase structural gene amoa as a functional marker: Molecular fine-scale analysis of natural ammonia-oxidizing populations. *Appl Environ Microbiol* 1997;**63**:4704-12. <https://doi.org/10.1128/aem.63.12.4704-4712.1997>

4. Chen S, Zhou Y, Chen Y, Gu J. Fastp: An ultra-fast all-in-one fastq preprocessor. *Bioinformatics* 2018;**34**:i884-i90. <https://doi.org/10.1093/bioinformatics/bty560>

5. Magoc T, Salzberg SL. Flash: Fast length adjustment of short reads to improve genome assemblies. *Bioinformatics* 2011;**27**:2957-63. <https://doi.org/10.1093/bioinformatics/btr507>

6. Edgar RC. Uparse: Highly accurate otu sequences from microbial amplicon reads. *Nat Methods* 2013;**10**:996-8. <https://doi.org/10.1038/nmeth.2604>

7. Wang Q, Garrity GM, Tiedje JM, Cole JR. Naive bayesian classifier for rapid assignment of rrna sequences into the new bacterial taxonomy. *Appl Environ Microbiol* 2007;**73**:5261-7. <https://doi.org/10.1128/AEM.00062-07>

8. Souza TP, Marques SC, da Silveira e Santos DM, Dias ES. Analysis of thermophilic fungal populations during phase ii of composting for the cultivation of agaricus subrufescens. *World J Microbiol Biotechnol* 2014;**30**:2419-25. <https://doi.org/10.1007/s11274-014-1667-3>

9. Callahan BJ, McMurdie PJ, Rosen MJ, Han AW, Johnson AJ, Holmes SP. Dada2: High-resolution sample inference from illumina amplicon data. *Nature Methods* 2016;**13**:581-3. <https://doi.org/10.1038/nmeth.3869>

10. Bolyen E, Rideout JR, Dillon MR, Bokulich NA, Abnet CC, Al-Ghalith GA. et al. Reproducible, interactive, scalable and extensible microbiome data science using qiime 2. *Nat Biotechnol* 2019;**37**:852-57. <https://doi.org/10.1038/s41587-019-0209-9>

11. Nilsson RH, Larsson KH, Taylor AFS, Bengtsson-Palme J, Jeppesen TS, Schigel D. et al. The unite database for molecular identification of fungi: Handling dark taxa and parallel taxonomic classifications. *Nucleic Acids Research* 2019;**47**:D259-D64. <https://doi.org/10.1093/nar/gky1022>

12. White TJ, Bruns T, Lee S, Taylor J Amplification and direct sequencing of fungal ribosomal rna genes for phylogenetics. In: Innis MA, Gelfand DH, Sninsky JJ *et al.* (eds.). *Pcr protocols*, San Diego: Academic Press. 315-22. Retreived from <https://www.sciencedirect.com/science/article/pii/B9780123721808500421>

13. Li D, Liu CM, Luo R, Sadakane K, Lam TW. Megahit: An ultra-fast single-node solution for large and complex metagenomics assembly via succinct de bruijn graph. *Bioinformatics* 2015;**31**:1674-6. <https://doi.org/10.1093/bioinformatics/btv033>

14. Wu YW, Simmons BA, Singer SW. Maxbin 2.0: An automated binning algorithm to recover genomes from multiple metagenomic datasets. *Bioinformatics* 2016;**32**:605-7. <https://doi.org/10.1093/bioinformatics/btv638>

15. Chklovski A, Parks DH, Woodcroft BJ, Tyson GW. Checkm2: A rapid, scalable and accurate tool for assessing microbial genome quality using machine learning. *Nature Methods* 2023;**20**:1203-12. <https://doi.org/10.1038/s41592-023-01940-w>

16. Chaumeil PA, Mussig AJ, Hugenholtz P, Parks DH. Gtdb-tk v2: Memory friendly classification with the genome taxonomy database. *Bioinformatics* 2022;**38**:5315-16. <https://doi.org/10.1093/bioinformatics/btac672>

17. Aramaki T, Blanc-Mathieu R, Endo H, Ohkubo K, Kanehisa M, Goto S. et al. Kofamkoala: Kegg ortholog assignment based on profile hmm and adaptive score threshold. *Bioinformatics* 2020;**36**:2251-52. <https://doi.org/10.1093/bioinformatics/btz859>

18. Quast C, Pruesse E, Yilmaz P, Gerken J, Schweer T, Yarza P. et al. The silva ribosomal rna gene database project: Improved data processing and web-based tools. *Nucleic Acids Res* 2013;**41**:D590-6. <https://doi.org/10.1093/nar/gks1219>

19. Kopylova E, Noe L, Touzet H. Sortmerna: Fast and accurate filtering of ribosomal rnas in metatranscriptomic data. *Bioinformatics* 2012;**28**:3211-7. <https://doi.org/10.1093/bioinformatics/bts611>

20. Patro R, Duggal G, Love MI, Irizarry RA, Kingsford C. Salmon provides fast and bias-aware quantification of transcript expression. *Nat Methods* 2017;**14**:417-19. <https://doi.org/10.1038/nmeth.4197>

21. Wagner GP, Kin K, Lynch VJ. Measurement of mrna abundance using rna-seq data: Rpkm measure is inconsistent among samples. *Theory Biosci* 2012;**131**:281-5. <https://doi.org/10.1007/s12064-012-0162-3>

22. Liu W, Russel J, Roder HL, Madsen JS, Burmolle M, Sorensen SJ. Low-abundant species facilitates specific spatial organization that promotes multispecies biofilm formation. *Environ Microbiol* 2017;**19**:2893-905. <https://doi.org/10.1111/1462-2920.13816>

23. BK M, K W, V U, BE R, DA S. Phylogenetic probes for analyzing abundance and spatial organization of nitrifying bacteria. *Appl Environ Microbiol* 1996;**62**:2156-62. <https://doi.org/10.1128/aem.62.6.2156-2162.1996>

24. Nguyen LT, Schmidt HA, von Haeseler A, Minh BQ. Iq-tree: A fast and effective stochastic algorithm for estimating maximum-likelihood phylogenies. *Mol Biol Evol* 2015;**32**:268-74. <https://doi.org/10.1093/molbev/msu300>

25. Letunic I, Bork P. Interactive tree of life (itol) v5: An online tool for phylogenetic tree display and annotation. *Nucleic Acids Res* 2021;**49**:W293-W96. <https://doi.org/10.1093/nar/gkab301>

26. Jain C, Rodriguez RL, Phillippy AM, Konstantinidis KT, Aluru S. High throughput ani analysis of 90k prokaryotic genomes reveals clear species boundaries. *Nat Commun* 2018;**9**:5114. <https://doi.org/10.1038/s41467-018-07641-9>

27. Buchfink B, Reuter K, Drost HG. Sensitive protein alignments at tree-of-life scale using diamond. *Nat Methods* 2021;**18**:366-68. <https://doi.org/10.1038/s41592-021-01101-x>
